# Determination of Eight Benzene Homologs in Ambient Air and Indoor Air in Residential Areas by Secondary Thermal Desorption–Gas Chromatography

**DOI:** 10.1155/ianc/9400881

**Published:** 2026-01-31

**Authors:** Tao Liu, Xiaoxue Yuan, Tiankuo Yang, Changxiao Yang, Hui Zhao, Jiwen Jiang, Jun Feng

**Affiliations:** ^1^ Civil Aviation High Altitude Aviation Medicine Research Center, Civil Aviation Flight University of China, Guanghan, 618307, Sichuan, China, cafuc.edu.cn; ^2^ Sichuan Center for Disease Control and Prevention, Chengdu, 610041, Sichuan, China; ^3^ College of Airport, Civil Aviation Flight University of China, Guanghan, 618307, Sichuan, China, cafuc.edu.cn

**Keywords:** air pollution analysis, ambient air, benzene homologs, gas chromatography, indoor air, secondary thermal desorption

## Abstract

This study developed and comprehensively validated an optimized analytical method based on secondary thermal desorption–gas chromatography (STD‐GC) for the simultaneous determination of eight key benzene homologs in ambient and indoor air of residential areas. Compared to solvent desorption and single‐stage thermal desorption, STD demonstrates superior desorption efficiency, reduced matrix interference, fully automated rapid operation, complete analyte transfer, thereby eliminating analyte losses while enhancing analytical accuracy and sensitivity, and solvent‐free ensuring friendly to human and the environment. Aiming at the characteristics of target compounds and complex environmental matrices (e.g., humidity, coexisting interferents), sampling parameters, thermal desorption conditions, and gas chromatographic separation conditions were systematically optimized, with particular emphasis on ensuring baseline separation of xylene isomers. The method demonstrated linearity over 10–1000 ng with correlation coefficients (*R* ^2^) > 0.992. The spiked recoveries of the method ranged between 90.5% and 117.3%, with relative standard deviations ranging between 0.8% and 9.2%, the detection limits ranged from 50 to 120 ng/m^3^, and the lower limits of quantitation ranged from 20 to 480 ng/m^3^. This study established a standardized and highly reliable analytical workflow, addressing the issues of parameter inconsistency and insufficient validation in existing methods when applied to paired residential‐indoor air studies. Using this method, ambient and indoor air samples were synchronously collected and analyzed in residential areas. Results demonstrated significant correlations between indoor and outdoor concentrations of benzene homologs. This study also provides a methodological foundation and practical guidance for accurate assessment of residents’ exposure to benzene homologs.

## 1. Introduction

With advances in modern industry, benzene homologs have become indispensable raw materials in chemical manufacturing [1–4]. These include eight priority compounds: benzene, toluene, ethylbenzene, *p*‐xylene, *m*‐xylene, *o*‐xylene, styrene, and *p*‐tert‐butyltoluene. Benzene is a known carcinogen, while the others demonstrate hepatotoxic, nephrotoxic, and neurotoxic effects in humans and aquatic organisms [5–10]. Indoor air pollution by benzene homologs (second only to formaldehyde) originates primarily from paints, coatings, adhesives, and synthetic building materials [11, 12]. Urbanization has exacerbated exposure risks as residential areas increasingly overlap with industrial zones. Consequently, monitoring these compounds in indoor and residential environments is critical. China’s 2019 National Health Commission initiative on indoor environmental health highlighted analytical challenges posed by ultralow concentrations (ng/m^3^range) in residential areas [6, 13]. This necessitates sensitive analytical methods for pollution assessment and mitigation.

Current methods for benzene homologs determination include solvent desorption/one‐stage thermal desorption–gas chromatography (GC) [14–16], solvent desorption/direct injection‐portable GC/mass spectrometry [17–21], solid phase (micro) extraction‐GC/mass spectrometry [22–27], needle trap extraction‐GC/mass spectrometry [28–30], differential absorption spectroscopy [31, 32], cataluminescence [33, 34], gas sensor [35, 36], and fluorimetry [37]. These sample preparation techniques are part of a broader trend toward solvent‐free, sensitive, and automated methods for volatile organic compound analysis in various matrices [38]. While solvent desorption‐GC remains widely used, it suffers from low sensitivity (due to 1.0 μL injection from a 1.0 mL desorption volume) and limitations in high‐humidity environments. Carbon disulfide, the desorption solvent, is hazardous and may contain benzene impurities. In addition, to use this desorption method, activated carbon will be used for sampling, which is strongly hydrophilic and thus not suitable for sampling in high‐humidity environments. For these reasons, this method is being gradually replaced by other methods. Direct injection and optical methods are suitable for the determination of high‐concentration benzenes in air in workplaces but are not sufficiently sensitive for the determination of benzene homologs in indoor air; additionally, they are costly and cannot be easily popularized. Cataluminescence and fluorimetry are highly sensitive and expected to be extensively used in the future and even employed as a new national standard method for rapid detection.

Secondary thermal desorption (STD) addresses these limitations by using Tenax‐TA tubes (hydrophobic polymer resin), incorporating a two‐stage desorption system with cryofocused analyte transfer and eliminating solvents while enhancing sensitivity through analyte focusing. Currently, the national standards have been established for the determination of benzene or benzene homologs that typically employs solvent desorption or one‐stage thermal desorption. STD has been reported relatively infrequently [39]. Herein, we establish an STD‐GC method for the simultaneous determination of eight benzene homologs in residential and indoor air. This method provides a reference for the revision of relevant national standards and the setting of exposure thresholds, ultimately contributing to public health protection.

## 2. Materials and Methods

### 2.1. Instruments and Reagents

The following instruments were used: a 7890A gas chromatograph equipped with a hydrogen flame ionization detector (Agilent Technologies, USA); a hydrogen generator (Peak, UK); a Unity Series 2 secondary desorber (Markes, UK); a LAB‐T220 aging instrument (Guangzhou Appinno Instrument Co., Ltd., China); Tenax‐TA adsorption tubes (Markes, UK); a DB‐WAX capillary column (30 m × 0.25 mm × 0.25 μm); a gas preparation instrument for preparing standard series by liquid external standard method (equipped with a high‐purity nitrogen flow regulator); an air sampling pump (flow range: 0.1–1.0 L/min, Wuhan Tianhong Instruments Co., Ltd., China); a multirange electronic soap film flow meter (Beijing Municipal Institute of Labour Protection, China); pipettes with volume ranges between 0.5 and 1000 μL (Biohit, Finland); and 10.0 μL microinjectors (Shimadzu, Japan).

A certified mixed standard solution of eight benzene homologs in methanol (benzene, toluene, ethylbenzene, *p*‐xylene, *m*‐xylene, *o*‐xylene, styrene, and *p*‐tert‐butyltoluene, 1000 ng/μL) was purchased from Beijing Tanmo Quality Testing Technology Co., Ltd, and methanol was purchased from Fisher Scientific (USA).

### 2.2. Sample Collection

After conditioning a Tenax‐TA sorbent tube (aged using a LAB‐T220 aging instrument), it was connected to the air inlet of a sampling pump. The sampling system flow rate was first calibrated with a multirange electronic soap film flow meter. Air sampling was conducted for 60 min at 0.1 L/min, with ambient temperature and atmospheric pressure recorded for standard volume conversion; a blank sample was collected concurrently. Sampling sites were positioned to avoid air vents and obstacles. Ambient air sampling sites were set on roofs of residential buildings near industrial areas; indoor air sampling sites were set ≥ 0.5 m from walls. Sampling sites were set at a height equal to the height of the human breathing zone, and heights were between 0.5 and 1.5 m. Doors and windows were closed for 12 h prior to sampling and kept closed throughout the process of sampling. The sampling duration should cover intervals with the worst ventilation. Residential areas were generally enclosed communities, so per building, two diagonal sampling sites were set. The number of indoor air sampling sites depends on the indoor area, with two sites set for a room with an area of < 50 m^2^, four for a room with an area of 50–100 m^2^, and six for a room with an area of > 100 m^2^, and all sampling sites should be set on diagonal lines or uniformly quincunx pattern. After sample collection, the sorbent tube was removed, sealed at both ends, placed in a desiccator containing activated charcoal, stored at 4°C in an organic reagent‐free refrigerator, and analyzed within 20 days. All parallel samples were analyzed individually by STD‐GC without pooling. The final concentration for each sampling point was the arithmetic mean of the detected values of all parallel samples collected during the four sampling events conducted over 2 weeks.

### 2.3. STD and Instrumental Analysis Conditions

**FIGURE 1:**
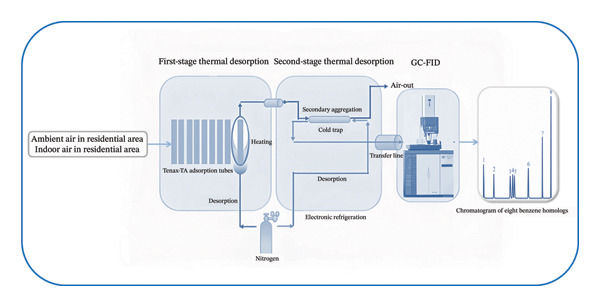
Schematic diagram of the STD‐GC system.

As shown in Figure 1, the sorbent tube was mounted on the desorber for STD under a splitless mode at a nitrogen desorption flow rate of 80 mL/min, with the temperature increased at a rate of 100°C/min from room temperature to 320°C and held at 320°C for 10 min. Cold trapping was performed under a split mode with a split ratio of 20:1, with the cold trap temperature held at 0°C for 0.5 min, then increased at a rate of 100°C/s to 300°C (held for 5 min), while the valve and transmission line were maintained at 200°C.

Benzene homologs were separated on a DB‐WAX capillary column (30 m × 0.25 mm × 0.25 μm). The column temperature was held at 60°C for 7 min and then programmed at a rate of 30°C/min to 150°C, followed by a post‐run at 230°C for 10 min. The carrier gas flow rate was 1.0 mL/min, the FID temperature was 300°C, and the injection port temperature was 240°C.

### 2.4. Method Validation

#### 2.4.1. Preparation of Standards

Using a pipette, 10, 50, 100, 250, 500, and 1000 μL of mixed standard solution were transferred to sample vials and diluted with methanol to 1.0 mL to produce 10, 50, 10, 250, 500, and 1000 ng/μL mixed standard working solutions. Standard curves were plotted using the liquid external standard method. Under an 80 mL/min nitrogen flow, an aged sorbent tube was connected to a liquid‐standard‐based gas preparation instrument. Using a microinjector, 1.0 μL of each standard concentration point was injected into the gas preparation instrument and purged for 5 min, followed by STD‐GC analysis. Analytes were identified by retention time. The liquid‐phase spiking is the core method for establishing the calibration curve of this study. The matrix conditions of methanol solvent and pure nitrogen flow in this method differ from those of real air samples (containing water vapor and trace impurities), which may exert a slight impact on the limit of detection (LOD). However, this limitation does not affect the detection applicability of the method for ng/m^3^‐level benzene homologs in residential air, and the LOD still meets the requirements of practical monitoring.

#### 2.4.2. Method Precision and Accuracy Tests

Within the standard linear range, low‐, medium‐, and high‐concentration mixed standards were separately added to six aged blank sorbent tubes and analyzed under experimental conditions described in Section 2.3, and relative standard deviations were calculated. Sampled sorbent tubes spiked with low‐, medium‐, and high‐concentration mixed standards were analyzed under the same conditions, and spike recoveries were calculated.

### 2.5. Quality Control

Used Tenax‐TA sorbent tubes should be aged at 320°C for 1.5 h in an aging instrument. Newly purchased sorbent tubes should be aged at 320°C for 3 h. Aged sorbent tubes should be placed in a desiccator and preserved in a refrigerator. Prior to sample collection, blank determinations were performed on 20% of the Tenax‐TA sorbent tubes. Analytes in blank tubes equivalent to a sample volume of 6 L were less than the limits of detection; otherwise, reaging should be performed. Prior to each determination, the chromatograph should be conditioned to remove any residue by programmed temperature increase. A reagent blank determination should be performed for each batch of reagents. A laboratory blank determination should be performed for each batch of samples. The experiment proceeded only when benzene homologs and other interfering substances in reagents and the laboratory environment are below the limits of detection. A blank sample should be collected for each sampling site, and the detected quantity of each analyte in the blank samples required to be < 10% of sample detections or equivalent to blank adsorbent tube results. Parallel samples constituted at least 10% in each batch of samples and the difference between measured values of parallel samples should not be greater than 20% of the mean value. A medium‐concentration point on the standard curve should be measured every 12 h for verification. The relative error between the measured value of the medium‐concentration verification point and the concentration of the corresponding point on the standard curve should ≤ 20%. Furthermore, meteorological parameters were systematically monitored during air sample collection. Specifically, we used a calibrated weather station installed near the sampling sites to record ambient temperature, relative humidity, and wind speed at 30‐min intervals. For indoor environments, temperature and humidity were measured simultaneously with sampling via portable sensors, ensuring alignment with the timing of outdoor data.

## 3. Results and Discussion

### 3.1. Optimization of Experimental Conditions

#### 3.1.1. Selection of Chromatographic Conditions

Considering that the injection port temperature could not be lower than the temperature of the thermal desorption transmission line, or the desorbed analytes would condense before entering the column and thus cannot be well separated, the effects of different injection port temperatures (200, 220, 240, 260, and 280°C) on the total peak area of the eight benzene homologs were investigated while keeping all other conditions constant. To further shorten the run time while ensuring baseline separation of all eight benzene homologs, a two‐factor three‐level orthogonal test was carried out, an approach consistent with methodologies used in chromatographic parameter optimization [40]. The results showed that, when the injection port temperature was 240°C, benzene homologs were vaporized to the maximum extent, and the largest total peak area was obtained. Since ethylbenzene, *p*‐xylene, *m*‐xylene, and *o*‐xylene could not be easily separated, the column temperature and flow rate will both have an impact on the separation effects and analytical run time. To further shorten the run time while ensuring the eight benzene homologs can be well separated, a two‐factor three‐level orthogonal test was carried out to optimize the column temperature program and the flow rate. The results showed that, when the column flow was maintained at 1.0 mL/min and the column temperature was programmed at a ramp of 30°C/min from an initial temperature of 60°C held for 7 min to 150°C, the eight benzene homologs could be well separated, and the total peak area was the largest while the analytical time was the shortest. Therefore, the above chromatographic conditions were selected for the analysis.

#### 3.1.2. Selection of STD Conditions

##### 3.1.2.1. First‐Stage Thermal Desorption Temperature

Boiling points of the eight benzene homologs vary significantly. Ethylbenzene, xylene, and, particularly, *p*‐tert‐butyltoluene (boiling point: 193°C) require a high desorption temperature, while the filler of Tenax‐TA adsorbent tubes requires the experimental temperature to be lower than 350°C. Therefore, the desorption effects of benzene homologs (based on the total peak area of the eight benzene homologs) using different desorption temperatures (240, 260, 280, 300, 320, and 340°C) were investigated. The results showed that the total peak area increased with the desorption temperature when it was below 320°C but plateaued at higher desorption temperatures. Therefore, the first‐stage desorption temperature was set as 320°C.

##### 3.1.2.2. Desorption Time

Unduly short desorption time would affect the desorption efficiency of benzene homologs. Moreover, some time is needed for increasing the desorption temperature to the set value. Thus, the effects of different desorption times (4, 6, 8, 10, and 12 min) on the total peak area of the eight benzene homologs were investigated. When the desorption time was 10 min, the total peak area was maximized. Therefore, a desorption time of 10 min was selected.

##### 3.1.2.3. Cold Trapping Temperature

Considering that the trapping and desorption efficiency of the eight benzene homologs varied at the same temperature, the effects of different cold trapping temperatures (−30, −20, −10, 0, 10, and 20°C) on the desorption efficiency were investigated. The total peak area was maximized at a cold trapping temperature of 0°C. Therefore, the optimal cold trapping temperature of 0°C was selected.

##### 3.1.2.4. Second‐Stage Thermal Desorption Temperature

To ensure benzene homologs were completely desorbed and other residues in the quartz tube were removed, the effects of different second‐stage desorption temperatures (280, 300, 320, and 340°C) on the desorption efficiency were investigated with the first‐stage desorption temperature (320°C), the desorption time (8 min), and the cold trapping temperature (0°C) kept constant. The results showed that the total peak area was the largest when the second‐stage desorption temperature was 300°C, and did not significantly change at higher temperatures. Thus, the second‐stage desorption temperature was set at 300°C.

### 3.2. Methodological Parameters

#### 3.2.1. Linearity, LOD, and Limit of Quantitation

**FIGURE 2:**
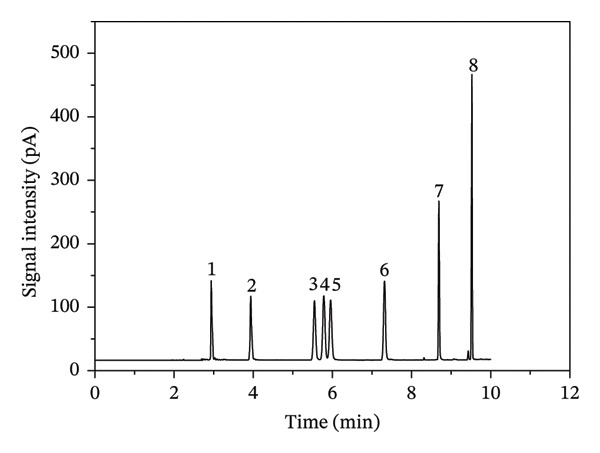
Chromatogram of standard solution of eight benzene homologs (1. benzene; 2. toluene; 3. ethylbenzene; 4. *p*‐xylene; 5. *m*‐xylene; 6. *o*‐xylene; 7. styrene; 8. *p*‐tert‐butyltoluene).

According to the method in Section 2.4.1, a chromatogram of the eight benzene homologs is shown in Figure 2. Figure 2 demonstrates that the eight benzene homologs were well separated. Standard curves of the eight benzene homologs were plotted with peak areas as the ordinate versus concentrations as the abscissa. The linear correlation coefficients of the eight benzenes were obtained. As indicated by the chromatogram, the xylene isomers achieved baseline separation with no peak overlap between adjacent peaks, and the resolution of all target compounds fully met the requirements for chromatographic quantification. For the eight benzene homologs, their retention times extended sequentially with the increase in boiling points, which was consistent with the retention law of hydrocarbon compounds on the polar DB‐WAX capillary column. The LOD was defined as three times the signal‐to‐noise ratio, and the limit of quantitation as four times the LOD, based on a sample volume of 6 L. The results are presented in Table 1. The eight benzene homologs show good linearity with correlation coefficients (*R* ^2^) > 0.992 in the range of 10–1000 ng, indicating reliable quantitative linearity. Limits of detection ranged between 50 and 120 ng/m^3^ (lowest for benzene: 50 ng/m^3^, highest for p‐tert‐butyltoluene: 120 ng/m^3^), and the limits of quantitation ranged between 200 and 480 ng/m^3^, which matches the retention behavior (high‐boiling compounds have slightly higher LODs due to stronger retention and slightly higher desorption difficulty), reflecting the method’s adaptability to target compounds with different boiling points.

**TABLE 1:** Linear equations, linear correlation coefficients, detection limits, and limits of quantitation.

Compound	Linear equations	Detection limits (ng/m^3^)	Limits of quantitation (ng/m^3^)
Benzene	*y* = 0.46189*x* + 21.37293	50	200
Toluene	*y* = 1.18912*x* − 23.46878	62	248
Ethylbenzene	*y* = 1.29624*x* − 18.32520	82	328
*p*‐Xylene	*y* = 1.22180*x* − 14.58682	79	316
*m*‐Xylene	*y* = 1.22028*x* − 14.02897	75	300
*o*‐Xylene	*y* = 1.28145*x* − 14.34973	84	336
Styrene	*y* = 1.28267*x* − 11.00490	115	460
*p*‐tert‐Butyltoluene	*y* = 1.24329*x* − 3.05662	120	480

#### 3.2.2. Thermal Desorption Efficiency and Stability Test

Using a 10‐μL microinjector, 1.0 μL of 250 ng/μL standard solution was injected into the gas preparation instrument (ultrapure nitrogen flow rate: 80 mL/min), and the analytes were collected onto six aged Tenax‐TA sorbent tubes. Desorption efficiency test results are presented in Table 2. The desorption efficiency of the eight benzene homologs ranged between 91.7% and 109.5%, with RSDs between 0.9% and 8.7%, indicating that under optimized conditions, the eight benzene homologs collected on Tenax‐TA sorbent tubes can be completely desorbed. Twelve aged Tenax‐TA sorbent tubes were prepared as described and stored in a 4‐°C refrigerator. On days 5, 10, 15, and 20, three sorbent tubes were taken and analytes were determined by STD‐GC. The results showed that the contents of the eight benzene homologs were stable over the 20 days of storage of the Tenax‐TA sorbent tubes in the refrigerator, the loss rate being within 10% at all time points. The results indicate that, with the Tenax‐TA sorbent tubes stored under closed and low‐temperature conditions, the eight benzene homologs are stable. Such factors as physical diffusion have no significant. Samples of the eight benzene homologs collected on Tenax‐TA sorbent tubes can be stored for 20 days under the above conditions. The thermal desorption efficiency and stability are superior to the national standard method and other reported methods.

**TABLE 2:** Desorbed efficiency and stability of eight benzene homologs.

Compound	Desorbed efficiency (%)	RSD (%, *n* = 6)	Measured value (ng)			
			5 d	10 d	15 d	20 d
Benzene	91.7	7.7	241	235	240	228
Toluene	95.6	8.7	244	234	230	234
Ethylbenzene	98.5	3.3	250	251	243	242
*p*‐Xylene	100.4	4.9	248	252	238	239
*m*‐Xylene	103.6	1.0	246	244	251	245
*o*‐Xylene	107.3	2.4	247	252	239	243
Styrene	99.8	1.4	247	249	254	240
*p*‐tert‐Butyltoluene	109.5	0.9	253	242	246	248

#### 3.2.3. Penetration Volume Determination

Tenax‐TA filler is a 2,6‐diphenylfuran porous polymer resin, which efficiently adsorbs and thermally desorbs hydrocarbons. In using Tenax‐TA for sampling, breakthrough is indicated when the analyte concentration in the outflow gas is 5% of the inflow gas. In actual determination, two sorbent tubes may be connected in tandem to the sampling pump, and the sampled volume when the content in the back tube accounts for 10% of the total is referred to as the “penetration volume”. The sampled volume for all analytes should be controlled within the penetration volume [41]. Using two tandem sorbent tubes, air samples of 2, 4, 6, and 7 L were collected. After sampling, the two tubes were connected to the gas outlet of the gas preparation instrument, and the contents of the eight benzene homologs in the front tube and the back tube were determined as described for the preparation of standard sample tubes. None of the eight benzene homologs was detected from the back tube when the sampled air volume was 2, 4 or 6 L, whereas benzene and toluene were detected from the back tube when the sampled air volume was 7 L. Thus, the secure sample volume under residential air concentration conditions is 6 L. Notably, the 6‐L safe sampling volume is specifically applicable to residential air scenarios. Accordingly, for applications in industrial or highly polluted areas, we recommend reducing the sampling volume or employing high‐capacity sorbents (e.g., multibed sorbent tubes) to prevent sorbent tube saturation.

#### 3.2.4. Method Precision, Accuracy, and Selectivity

According to the method in Section 2.4.2, results of the method precision and accuracy are presented in Table 3. Table 3 demonstrates that spiked recoveries of this method ranged from 90.5% to 117.3%, with relative standard deviations ranging between 0.8% and 9.2%. Recovery rates exceeding 110% may be attributed to systematic errors in calibration curves or those associated with the processes of liquid‐phase spiking and gas‐phase sampling. Furthermore, three common coexisting interferents (formaldehyde, acetone, ethanol) in residential ambient air were selected to evaluate the selectivity of the method. Certain concentrations of formaldehyde, acetone, and ethanol were simultaneously spiked into real air samples, which were then analyzed under the same thermal desorption and instrumental analysis conditions. The results showed that eight benzene homologs were completely separated from the interferents, with no peak overlapping or co‐elution observed, demonstrating that the analytical method has good selectivity.

**TABLE 3:** Recoveries and relative standard deviations of eight benzene homologs.

Compound	Recoveries (%)			Relative standard deviations (%)		
	10 ng/μL	250 ng/μL	1000 ng/μL	50 ng/μL	250 ng/μL	1000 ng/μL
Benzene	90.5	99.8	94.6	8.4	6.4	7.1
Toluene	95.2	96.7	101.2	9.2	2.6	3.9
Ethylbenzene	102.1	98.8	104.2	1.1	4.9	0.8
*p*‐Xylene	107.5	111.7	103.7	6.3	1.6	4.4
*m*‐Xylene	105.6	99.9	112.4	1.2	6.8	1.7
*o*‐Xylene	97.9	114.2	108.3	5.1	0.9	4.9
Styrene	117.3	98.6	114.9	0.8	1.5	5.5
*p*‐tert‐Butyltoluene	105.8	115.8	109.5	7.1	4.2	3.6

### 3.3. Sample Analysis and Concentration Trends

In March and August 2024, air samples were collected from two ambient air sampling points (AASP) and two indoor air sampling points (IASP) (two parallel samples per point every three days, two blank samples every three days) and analyzed by the established method. Monthly mean concentrations and total concentrations of the eight benzene homologs were calculated. The results are presented in Table 4. Table 4 shows that concentrations of benzene homologs (particularly benzene and toluene, which have low boiling points) in August were significantly higher than in March. In March, contents of benzene homologs were higher in IASP air than in AASP air, whereas in August, contents of benzene homologs were higher in AASP air than in IASP air. This is mainly because benzene homologs contained in indoor building and decoration materials, office equipment, household appliances, and other articles are prone to volatilize into indoor air. March features low air temperature and frequent hazy weather, so benzene homologs in ambient air in the industrial areas will not easily diffuse to residential areas, while, in August, the outdoor temperature is high, so benzene homologs in industrial areas will easily diffuse to residential areas and then to indoor air. Although we did not conduct direct on‐site measurement of industrial source emissions, the aforementioned results regarding the easy diffusion effect of industrial zones are consistent with the spatial concentration gradients of industrial zones and the monitoring data from regional environmental stations. Additionally, ambient air in industrial areas is much more seriously polluted by benzene homologs than indoor air. At all sampling sites, the concentrations of high‐boiling benzene homologs (styrene and *p*‐tert‐butyltoluene) were low. This is because benzene homologs with high boiling points are preferentially adsorbed by solid‐phase particles in air.

**TABLE 4:** Average and total concentrations of eight benzene homologs in ambient air and indoor air (ng/m^3^).

Compound	March				August			
	AASP1	IASP1	AASP2	IASP2	AASP1	IASP1	AASP2	IASP2
Benzene	747	4457	1211	7355	14,677	12,990	31,230	22,344
Toluene	758	3245	2003	6988	15,098	10,342	30,098	20,003
Ethylbenzene	598	2098	1978	5123	13,401	8245	28,890	18,930
*p*‐Xylene	487	1876	1554	3466	8890	5600	27,334	17,945
*m*‐Xylene	681	2334	1799	4001	9866	4968	27,899	19,023
*o*‐Xylene	594	2101	1823	3877	8034	5021	26,580	16,506
Styrene	161	ND^1^	356	194	657	489	2678	1231
*p*‐tert‐Butyltoluene	137	ND	588	145	324	ND	ND	ND
∑eight benzene homologs	4163	16,111	11,312	31,149	70,947	45,166	174,709	115,982

^1^ND: not detected.

In addition, to evaluate the impact of meteorological variables (humidity, wind speed, temperature) on concentration fluctuations of benzene homologs between indoor and outdoor environments, we also conducted relevant analyses. The results indicated that higher outdoor temperatures (≥ 30°C) were associated with increased indoor benzene homolog concentrations, which may be related to the potential enhancement of building material volatility and elevated ventilation rates. Conversely, elevated wind speeds (> 2 m/s) were associated with reduced outdoor benzene homolog concentrations, while exerting minimal influence on indoor levels—a pattern potentially linked to wind‐driven dispersion in outdoor environments and the barrier effect of building envelopes indoors. When indoor humidity exceeded 70%, concentrations of polar homologs (e.g., xylene) decreased slightly, possibly resulting from competitive adsorption between water vapor and target compounds on indoor surfaces. This effect was less pronounced outdoors due to the greater air volume dilution in outdoor environments. Although these variables were not controlled in this study (consistent with real‐world monitoring scenarios), systematic monitoring of them provided a reliable basis for interpreting the data on outdoor vs. indoor differences.

## 4. Conclusions

This study successfully established and rigorously validated a standardized analytical method based on STD‐GC for simultaneous and high‐precision analysis of eight benzene homologs in ambient and indoor air of residential areas. Through targeted optimization and systematic validation of sampling, thermal desorption, and chromatographic separation, the method demonstrates excellent performance in sensitivity, precision, accuracy, and interference resistance (e.g., humidity), providing a solid guarantee for reliable determination of target benzene homologs in complex environmental matrices. Additionally, the method overcomes the desorption challenge of high‐boiling homologs (e.g., p‐tert‐butyltoluene), achieving desorption efficiency of 91.7%–109.5%, higher than conventional methods; Tenax‐TA tubes allow 6 L sampling without breakthrough, and samples remain stable for 20 days at 4°C (loss rate < 10%), enabling large‐scale field sampling, whereas solvent desorption requires analysis within 24 h; field applications reveal seasonal concentration fluctuations (e.g., 40% higher outdoor levels in August vs. March).

Compared with the methods reported previously, this study provides a fully optimized and comprehensively validated, complete, and reproducible analytical protocol specifically tailored for target benzene homologs and residential/indoor air scenarios. This standardized method was systematically applied for the first time to paired sampling studies of outdoor–indoor air in residential areas, observing concentration patterns and suggesting potential source influences between them. This standardized approach is not only conducive to significantly enhancing the comparability and reliability of data quality but also offers valuable technical reference for potential large‐scale population exposure assessments, the setting of exposure thresholds for unregulated homologs, and related environmental health risk studies in the future.

## Funding

This work was financially supported by Science and Technology Projects of Tibet Autonomous Region (XZ202401ZY0016), the Fundamental Research Funds for the Central Universities (24CAFUC03086), Educational Reform Project of Civil Aviation Flight University of China (202521), Sichuan Province Science and Technology Support Program (2023YFQ0068, 2023ZYD0152), and Sichuan Center for Disease Control and Prevention Research Project (ZX202106).

## Conflicts of Interest

The authors declare no conflicts of interest.

## Data Availability

Data sharing is not applicable to this article as no datasets were generated or analyzed during the current study.
